# A Visceral Æsthesio-Neurosis

**Published:** 1878-09

**Authors:** James I. Tucker

**Affiliations:** Chicago


*A Visceral Æsthesio-Neurosis.*

As a sequel to enteric disease, it has twice lately fallen to my lot to observe a distressing and tenacious symptom which may properly be classed among the visceral æsthesio-neuroses. It is a hyperalgesia and a dysæsthesia referred to the region of the umbilicus, but radiating probably from the nerves which preside over the excretory function of the colon, to be finally interpreted by the higher centers. The first case was that of a physician who had suffered from colitis ; the second that of a lady 60 years of age, who had passed through typhoid fever. The primary disease in both cases occurred between six and seven years ago. So close is the resemblance between the two cases that I venture to report them together. There is abnormal sensibility in the large intestine, and pain is the inevitable consequence of even a small fœcal accumulation, which calls into play the reflex action. As the morning approaches, the sleep is disturbed, and at five o'clock precisely, and daily (with only an occasional intermission, sometimes lasting for weeks), the pain sets in. The pain continues until there is an evacuation at about seven, and then gradually dies away. It does not recur in the daytime, except under the influence of sudden change in atmospheric pressure, of mental emotion, fatigue, indigestion, etc. Its periodicity and its tendency to excitation under psychic and nervous influences, shows its character to be essentially neurotic. There is doubtless some slight organic alteration of the mucous surfaces—the terminal ends of the nerves infringed upon or altered, so as, in a measure, to interfere with normal conduction of impressions. Under unusually exciting conditions, there is a free discharge of mucus. As in hepatic and enteric disturbances, generally, there is in these cases a degree of cerebro-hyposthenia and the patients are unduly anxious about themselves. It is to be lamented that they have both resorted to opium as the only "stayer of anguish." But I am gradually getting them to rely on other measures, and I have done much already by psycho-physical methods, by regimen, by tincture of colocynth and the surreptitious substitution of a *placebo* for the narcotic which in my opinion the circumstances amply justify.

JAMES I. TUCKER, M. D., *Chicago.*

